# Supplementation with Phycocyanobilin, Citrulline, Taurine, and Supranutritional Doses of Folic Acid and Biotin—Potential for Preventing or Slowing the Progression of Diabetic Complications

**DOI:** 10.3390/healthcare5010015

**Published:** 2017-03-14

**Authors:** Mark F. McCarty

**Affiliations:** Catalytic Longevity, 7831 Rush Rose Dr., Apt. 316, Carlsbad, CA 92009, USA; markfmccarty@gmail.com; Tel.: +1-760-216-7272

**Keywords:** diabetic complications, NADPH oxidase, endothelial nitric oxide synthase, nitric oxide, phycocyanobilin, citrulline, taurine, folic acid, biotin

## Abstract

Oxidative stress, the resulting uncoupling of endothelial nitric oxide synthase (eNOS), and loss of nitric oxide (NO) bioactivity, are key mediators of the vascular and microvascular complications of diabetes. Much of this oxidative stress arises from up-regulated nicotinamide adenine dinucleotide phosphate (NADPH) oxidase activity. Phycocyanobilin (PhyCB), the light-harvesting chromophore in edible cyanobacteria such as spirulina, is a biliverdin derivative that shares the ability of free bilirubin to inhibit certain isoforms of NADPH oxidase. Epidemiological studies reveal that diabetics with relatively elevated serum bilirubin are less likely to develop coronary disease or microvascular complications; this may reflect the ability of bilirubin to ward off these complications via inhibition of NADPH oxidase. Oral PhyCB may likewise have potential in this regard, and has been shown to protect diabetic mice from glomerulosclerosis. With respect to oxidant-mediated uncoupling of eNOS, high-dose folate can help to reverse this by modulating the oxidation status of the eNOS cofactor tetrahydrobiopterin (BH4). Oxidation of BH4 yields dihydrobiopterin (BH2), which competes with BH4 for binding to eNOS and promotes its uncoupling. The reduced intracellular metabolites of folate have versatile oxidant-scavenging activity that can prevent oxidation of BH4; concurrently, these metabolites promote induction of dihydrofolate reductase, which functions to reconvert BH2 to BH4, and hence alleviate the uncoupling of eNOS. The arginine metabolite asymmetric dimethylarginine (ADMA), typically elevated in diabetics, also uncouples eNOS by competitively inhibiting binding of arginine to eNOS; this effect is exacerbated by the increased expression of arginase that accompanies diabetes. These effects can be countered via supplementation with citrulline, which efficiently enhances tissue levels of arginine. With respect to the loss of NO bioactivity that contributes to diabetic complications, high dose biotin has the potential to “pinch hit” for diminished NO by direct activation of soluble guanylate cyclase (sGC). High-dose biotin also may aid glycemic control via modulatory effects on enzyme induction in hepatocytes and pancreatic beta cells. Taurine, which suppresses diabetic complications in rodents, has the potential to reverse the inactivating impact of oxidative stress on sGC by boosting synthesis of hydrogen sulfide. Hence, it is proposed that concurrent administration of PhyCB, citrulline, taurine, and supranutritional doses of folate and biotin may have considerable potential for prevention and control of diabetic complications. Such a regimen could also be complemented with antioxidants such as lipoic acid, N-acetylcysteine, and melatonin—that boost cellular expression of antioxidant enzymes and glutathione—as well as astaxanthin, zinc, and glycine. The development of appropriate functional foods might make it feasible for patients to use complex nutraceutical regimens of the sort suggested here.

## 1. NADPH Oxidase, Uncoupled eNOS, and Decreased NO Bioactivity Mediate Diabetic Complications

Oxidative stress, and the disruption of nitric oxide production and bioactivity which this entails, are believed to be key mediators of the complications of diabetes. Although increased mitochondrial superoxide production in glucose-permeable tissues can contribute to this oxidative stress, up-regulation of NADPH oxidase activity and uncoupled nitric oxide synthase are major culprits in this regard [1,2,3,4,5,6,7,8,9,10,11,12,13,14,15]. The hyperglycemia and, in type 2 diabetics, excessive free fatty acid levels characteristic of diabetes can stimulate NADPH oxidase activity via increased diacylglycerol synthesis and subsequent activation of protein kinase C [1]. In adipocytes, activation of toll-like receptor 4 by saturated fatty acid/fetuin-A complexes stimulates NADPH oxidase activity, contributing to adipocyte insulin resistance and aberrant production of adipokines typical of type 2 diabetes [16,17,18]. Moreover, interaction of advanced glycation end products (AGEs) with the receptor for AGEs (RAGE) receptor triggers activation of NADPH oxidase; there is strong reason to suspect that the resulting oxidative stress is a key mediator of the diabetic complications driven by AGE exposure [2].

The ways in which oxidative stress and the associated decline in NO bioactivity promote diabetic complications are complex, and still being unraveled. In regard to glomerular damage in diabetic nephropathy, modulation of podocyte and mesangial cell function plays a key role. Podocytes express high activities of eNOS and soluble guanylate cyclase [19]. Exposure of these cells to hyperglycemia triggers activation of protein kinase C, which in turn induces expression of Nox4 [20]. The resulting oxidative stress lowers cGMP levels and protein kinase G (PKG) activity, and, as a result, podocytes produce and secrete less of the basement membrane proteins nephrin and podocin required for prevention of albuminuria [21]. This oxidative stress, if severe, can also trigger podocyte apoptosis. Hyperglycemia acts on mesangial cells to boost synthesis of latent TGF-beta. Activation of TGF-beta requires interaction with thrombospondin-1 (TSP1), and, under hyperglycemic conditions, PKG activity suppresses transcription of the TSP1 gene [22]. Hence, the loss of PKG activity in the diabetic glomerulus boosts TSP1 activity, which in turn promotes activation of latent TGF-beta; this hormone then induces glomerulosclerosis by stimulating mesangial cell production of fibronectin and collagen.

With respect to diabetic retinopathy, increased contraction of retinal microvascular pericytes contributes to the lessening of retinal perfusion that in turn evokes pathologenic neovascularization [23]. Pericytes express eNOS, soluble guanylate cyclase, and PKG, and NO/cGMP suppress the contraction of pericytes, as they do in vascular smooth muscle [23,24]. Hyperglycemia and advanced glycation end products (AGEs), via stimulation of NAPDH oxidase in pericytes, impair NO bioactivity and hence trigger pericyte contraction [25,26,27,28]. Moreover, this oxidative stress can also trigger pericyte apoptosis [26]. NADPH oxidase activation may play a more general role in AGE-mediated micro- and macrovascular complications of diabetes [2]. Defective repair of the retinal microvasculature also contributes to the genesis of diabetic retinopathy. CD34+ endothelial precursor cells (EPCs), originating in the bone marrow, migrate to sites of endothelial damage to promote repair. However, this protective mechanism is dysfunctional in diabetics [29]. EPCs express eNOS activity, and cGMP-mediated activation of PKG is essential for regulated migration of these cells [29,30]. Hyperglycemia triggers NADPH oxidase activity in EPCs, and this in turn uncouples eNOS and impairs PKG activity, inhibiting the migration of EPCs and thus impeding repair of damaged retinal capillaries [14,31,32]. This dysfunction of EPCs may also play a role in impaired wound healing characteristic of diabetes [33].

Dysfunction and apoptotic death of Schwann cells is believed to play a role in diabetic neuropathy [34]. Healthy Schwann cells aid survival of neighboring neurons by producing the trophic hormones nerve growth factor (NGF) and neurotrophin-3 (NT3). This protection is contingent on neuronal production of NO (via nNOS), which in turn promotes production of cGMP and activation of PKG in Schwann cells [35]. Hyperglycemia promotes oxidative stress in Schwann cells and neurons, which in turn could be expected to impede NO bioactivity; in addition, hyperglycemia boosts PDE5 activity in Schwann cells, which likewise lowers cGMP levels [36,37,38]. Oxidative stress and NO bioactivity might also influence diabetic neural function by modulating endoneurial blood flow, a decline of which plays a role in diabetic neuropathy. Hyperglycemic activation of NADPH oxidase in endothelial cells can impair endoneurial perfusion by impeding NO-mediated dilation of vascular smooth muscle [39].

The increased risk or macrovascular disease in diabetics likewise may reflect, in part, endothelial dysfunction stemming from NAPDH oxidase activation, eNOS uncoupling, and loss of NO bioactivity [9,40]. Loss of such bioactivity also appears to contribute to diabetic cardiomyopathy and platelet hyperaggregabilty [41,42].

Activation of NADPH oxidase in adipose tissue and pancreatic beta cells plays a mediating role in the insulin resistance and beta cell dysfunction characteristic of type 2 diabetes. Activation of NADPH oxidase in adipocytes and resident macrophages contributes to the inflammation that compromises adipocyte insulin sensitivity, which in turn leads to the excess flux of free fatty acids that promotes systemic insulin resistance and hyperlipidemia [1,18,43]. Furthermore, chronic excessive activation of NADPH oxidase in beta cells is a mediator of the failure of glucose-stimulated insulin secretion and of the beta cell apoptosis that collaborate with systemic insulin resistance to usher in overt diabetes [1,44,45,46,47,48,49,50].

Recent prospective epidemiology points to concurrent statin use as possibly protective with respect to diabetic retinopathy and neuropathy [51]. These findings are intriguing in light of the fact that potent doses of lipophilic statins have the potential to down-regulate the activity of certain NADPH oxidase complexes by inhibiting isoprenylation of Rac1 [52].

## 2. Phycocyanobilin: A Nutraceutical Inhibitor of NADPH Oxidase

There is good reason to suspect that phycocyanobilin (PhyCB), a light-harvesting chromophore of cyanobacteria (such as spirulina) that is a metabolite and homolog of biliverdin, can inhibit certain isoforms of NADPH oxidase in a manner analogous to bilirubin [53,54,55,56,57,58]. It is notable that diabetics with Gilbert syndrome—in which plasma levels of free bilirubin are chronically elevated—are only about a third as likely as other diabetics to develop nephropathy, retinopathy, or coronary disease [59]. Other epidemiology likewise links increased plasma bilirubin with reduced risk for these complications, as well as peripheral atherosclerosis and diabetic neuropathy [60,61,62,63,64,65,66,67,68,69,70,71,72,73,74,75,76,77,78,79,80]. Oral administration of either PhyCB or biliverdin has been shown to inhibit glomerular sclerosis and oxidative stress in diabetic mice [58,81]. Additionally, oral administration of either whole spirulina or of phycocyanin (the protein which contains PhyCB as a covalently-linked chromophore) has shown anti-atherosclerotic effects in rodent models of this disorder [82,83,84,85,86]. These findings correlate well with epidemiology correlating increased plasma bilirubin with decreased risk for atherogenesis [87,88,89,90].

With respect to the role of NADPH oxidase activation in the genesis of metabolic syndrome and type 2 diabetes, studies with rodent models of these syndromes report favorable effects of oral phycocyanin or whole spirulina on glycemic control, serum lipid profile, blood pressure, and steatohepatitis [91,92,93,94,95,96]. Also, two clinical trials, in which spirulina was administered (likely in suboptimal doses) to type 2 diabetics, likewise found modest improvements in these parameters [97,98]. Furthermore, epidemiological studies, some of them prospective, have found that increased serum bilirubin is associated with decreased risk for metabolic syndrome or type 2 diabetes [69,99,100,101,102,103,104,105,106]. Moreover, among patients who are already diabetic, serum bilirubin is reported to correlate inversely with HbA1c and duration of diabetes, and directly with C-peptide levels [107,108,109]. Oral administration of biliverdin, the bilirubin precursor, prevents or postpones beta cell failure in diabetes-prone db/db mice [110].

Concentrated preparations of PhyCB per se for nutraceutical use are not yet available. Doses of up to 1 g phycocyanin daily have achieved “generally recognized as safe” status from the U.S. Food and Drug Administration [111]. Spirulina has been a traditional food in some cultures, and rodents can ingest 30% of their calories from spirulina for 13 weeks without clear harm; much lower intakes exert a wide range of protective effects in rodent models of disease, and provide protection from many toxins [112,113,114]. Whereas it is certainly conceivable that a sufficiently high intake of concentrated PhyCB could notably compromise immune defenses, much lower intakes can be expected to have valuable clinical potential if humans assimilate and metabolize this compound like rodents do.

## 3. High-Dose Folate Combats eNOS Uncoupling

Oxidative stress impairs effective NO activity in several ways: oxidizing tetrahydrobiopterin; inhibiting dimethylarginine dimethylaminohydrolase (DDAH), and thereby boosting intracellular levels of the eNOS inhibitor/uncoupler asymmetric dimethylarginine (ADMA) [115,116,117,118,119]; and direct quenching of NO by superoxide, leading to production of the potent oxidant peroxynitrite. Peroxynitrite is a mediator of the oxidation of tetrahydrobiopterin; and it can also inhibit a key target of NO bioactivity, soluble guanylate cyclase (sGC), by oxidizing the ferrous iron in its attached heme group [120,121,122,123]. Oxidized sGC is not only unresponsive to NO, but it also is prone to lose its heme group, leading to its proteasomal degradation.

Tetrahydrobiopterin is a cofactor for endothelial nitric oxide synthase (eNOS). Dihydrobiopterin, its oxidation product, is a competitive inhibitor of tetrahydrobiopterin’s binding to eNOS, and a low ratio of tetrahydrobiopterin to dihydrobiopterin promotes eNOS uncoupling, such that eNOS becomes a source of superoxide [124,125]. High-dose folate can be expected to promote recoupling of this enzyme by increasing the ratio of tetrahydrobiopterin to dihydrobiopterin. When administered in supraphysiological doses, elevated levels of reduced metabolites of folate accumulate within vascular endothelium and other tissues [126]. These reduced metabolites have versatile oxidant scavenging activity—in particular, they scavenge products of peroxynitrite which oxidize tetrahydrobiopterin to dihydrobiopterin [126,127,128]. Moreover, these folate metabolites promote induction of the enzyme dihydrofolate reductase, an enzyme which participates not only in folate metabolism, but also reduces dihydrobiopterin to the tetrahydro form [126,129,130,131]. Hence, high-dose folate has potential for suppressing eNOS uncoupling both by slowing the rate of oxidation of tetrahydrobiopterin, and by promoting the reconversion of dihydrobiopterin to tetrahydrobiopterin. Favorable effects of high-dose folate (5 mg, three times daily) on oxidative stress in diabetics have been reported that may reflect improved function of eNOS, as well as the scavenging activities of reduced folates [132,133]. Intravenous administration of 5-methyltetrahydrofolate has been reported to achieve acute improvement of endothelium-dependent vasodilation in diabetics, likewise likely stemming from recoupling of eNOS [134,135,136]. Oral folate has improved diabetic endothelial function in some studies but not others; the negative studies employed doses no higher than 5 mg daily [135,136,137]. Kurt Oster, who pioneered the clinical use of high-dose folate for vascular health, employed and recommended a daily dose of 40–80 mg [138,139]. He reported that administration of high-dose folate was associated with rapid healing of a diabetic ulcer that previously had been refractory, likely reflecting a key role for NO in wound healing [140,141,142,143]. No evident adverse effects were seen with this regimen.

## 4. Citrulline Can Counter the Adverse Impact of ADMA and Arginase on eNOS Activity

eNOS can also generate superoxide when it fails to bind its substrate L-arginine [144,145,146]. Although intracellular concentrations of arginine are usually far higher than its binding constant to eNOS, cells generate an arginine metabolite, asymmetric dimethylarginine (ADMA), which has very high affinity for eNOS and acts as a competitive inhibitor of arginine’s binding [147]. This agent is actively transported into endothelial cells, which markedly amplifies its capacity to act as a competitive antagonist for arginine [148]. ADMA originates when arginine groups in intact proteins are methylated on their guanidino head groups by a group of enzymes known as “protein arginine N-methyltransferases” (PRMTs); “asymmetric” refers to the fact that, in ADMA, one of the two nitrogens in this head group is dimethylated, whereas the other remains unmethylated [149]. Free ADMA is subsequently liberated when the protein carrying it is proteolysed. An enzyme dedicated to degrading ADMA, dimethylarginine dimethylaminohydrolase (DDAH), is responsible for about 80% of ADMA turnover, and its activity is a major determinant of ADMA levels within cells [150,151].

The ratio of arginine to ADMA within cells is hence a key determinant of eNOS function. A high ratio is needed for effective NO production and minimal superoxide generation, whereas a low ratio can make eNOS a significant source of superoxide and a poor source of, N.O. This ratio can be lowered by the activity of intracellular arginase, which transforms arginine to ornithine [152]. The ratio of arginine to ornithine, in cells or systemically, can be used as an assessment of effective arginase activity [153].

Rodent and clinical studies, in the main, tend to conclude that diabetes is associated with increased plasma ADMA levels; moreover, within the vasculature, decreased DDAH activity and elevated arginase activity is observed [115,154,155,156,157,158,159,160,161,162,163]. The plasma ornithine/arginine ratio is elevated in type 2 diabetics, indicative of a global increase in arginase activity [153]. Oxidative stress is capable of reducing the expression and activity of DDAH, whereas arginase expression is stimulated by p38 MAP kinase—whose activity, in turn, is responsive to oxidative stress [160,162,164,165,166,167,168]. Hence oxidative stress works to lower the arginine/ADMA ratio, and the resulting increase in superoxide generation tends to compound this oxidative stress—a vicious cycle analogous to that which promotes oxidation of BH4. Clearly, measures which boost arginine levels have potential for normalizing eNOS activity and controlling oxidative stress in diabetics.

While supplemental arginine can be employed to enhance intracellular arginine/ADMA ratios, this strategy is complicated by the fact that inducible arginase activity in gut bacteria, the GI mucosa, and the liver degrade a large amount of administered arginine before it can reach the systemic circulation and the body’s tissues [169,170]. Arginine supplementation, as a support for eNOS activity, tends to become less effective over time owing to induction of this arginase activity. Counterintuitively, supplementation with citrulline, to which arginine is converted during coupled eNOS activity, is far more effective for raising tissue arginine levels [169,171,172]. The citrulline generated by eNOS is rapidly reconverted to arginine in a two-step reaction. When administered orally, citrulline escapes degradation by argininase (indeed, it is a competitive inhibitor of arginase activity), is absorbed efficiently, and, once taken up into cells, is quickly converted to arginine. So supplemental citrulline represents an efficient delivery form for intracellular arginine [169,172]. A further advantage of citrulline is that, as compared to arginine, it has a far milder flavor that makes feasible its administration in drinks or functional foods [173]. Curiously, the most potent food source of citrulline is watermelon juice, which provides about 1.3 g citrulline per liter [174,175].

Considerable prospective epidemiology implicates ADMA as an independent risk factor for cardiovascular events in the general population [173,176]. Several studies focused on diabetics, though not all [177], likewise find that ADMA is a negative prognostic factor for cardiovascular health [154,178,179,180,181,182]. Moreover, a number of case-control studies have reported higher ADMA levels in diabetics afflicted with nephropathy retinopathy, or neuropathy [183,184,185,186,187,188,189,190]. Higher ADMA also was found in diabetics with vertebral fractures, likely reflecting a role for eNOS in bone health [191,192]. Since ADMA may serve as a marker for oxidative stress, it is not entirely clear that ADMA is a mediating risk factor in these regards, but this seems likely in light of the role of diminished eNOS activity in the genesis of diabetic complications.

With respect to diabetic nephropathy, two out or three rodent studies conclude that supplemental arginine or citrulline can impede onset of this disorder; in one of these studies, citrulline but not arginine was effective [193,194]. Several studies report that supplemental arginine aids wound healing in diabetic rats, likely reflecting a key role for eNOS in the wound healing process [195,196,197]. In one recent study, joint supplementation with citrulline and a biosynthetic precursor of BH4, sepiaterin, had a favorable impact on the evolution of diabetic cardiomyopathy in obese diabetic mice; this supplementation also minimized infarct volume in diabetic and non-diabetic mice subjected to cardiac ischemia-reperfusion [198]. (A comparable effect might have been expected with citrulline and high-dose folate.) An alternative strategy, arginase inhibition or knock-out, has also been shown to confer benefits in diabetic rodents [160,163,199,200,201].

To date, clinical effects of citrulline supplementation in diabetics have received minimal attention. In other contexts, supplemental citrulline has been shown to confer clinical benefits in daily intakes of 3–6 g daily [173]. No adverse effects have been reported at these doses; gastrointestinal tolerance at these high doses reflects its efficient absorption.

## 5. Biotin Can “Pinch Hit” for NO in Activation of Soluble Guanylate Cyclase

The loss of NO bioactivity in certain diabetic tissues leads to decreased production of cyclic GMP (cGMP), as NO potently activates the soluble guanylate cyclase. Decreased production of cGMP, in turn, is thought to be a key mediator of diabetic complications—a view that is supported by the protective utility of phosphodiesterase 5 (PDE5) inhibitors in rodent models of diabetic nephropathy, neuropathy, and cardiomyopathy [21,35,38,202,203]. Likewise, drugs which directly activate sGC inhibit the progression of diabetic nephropathy and cardiomyopathy in rats [204,205].

In concentrations roughly one-hundred-fold higher than the physiological plasma level, the vitamin biotin directly activates sGC; the maximal activation achieved in this way is only two-three-fold, far less potent than the 100-fold enhancement of activity seen with optimal concentrations of NO [206,207,208]. The fact that the activation of sGC achieved with biotin is relatively modest likely explains why mega-doses of this vitamin are well tolerated—whereas excessive NO levels can induce profound hypotension. Children with biotin-responsive genetic disorders have taken 100 mg daily or more without evident adverse effects, and pilot trials with high-dose biotin in multiple sclerosis, employing 100 mg three times daily, have not been attended by important side effects aside from a low incidence of gastrointestinal discomfort that remits over time [209,210,211]. (However, clinicians should be aware that biotin doses of this magnitude can interfere with thyroid function tests, such that they incorrectly suggest hyperthyroidism [212]).

In rodent models of diabetes, high-dose biotin—likely via effects mediated by cGMP—acts on the liver to promote induction of glucokinase, while suppressing induction of enzymes which promote gluconeogenesis and lipogenesis [213,214,215,216,217]. When blood glucose is elevated, increased glucose flux through glucokinase exerts a feedback suppression of gluconeogenesis and hepatic glucose output that contributes to appropriate glucose tolerance; this mechanism also helps to moderate fasting glucose [218]. Biotin-mediated induction of glucokinase might be of particular benefit in type 1 diabetics, in whom hepatic insulin exposure and glucokinase expression is constantly subnormal despite subcutaneous insulin therapy [217,219,220,221]. In beta cells, biotin-stimulated cGMP synthesis likewise boosts glucokinase expression, helping to correct a down-regulation of glucokinase activity that plays a key role in the beta cell dysfunction characteristic of type 2 diabetes [222,223,224,225,226]. In both the liver and the kidney, glucokinase functions as a “glucose sensor”, and subnormal glucokinase activity results in the impaired control of gluconeogenesis and the failure of glucose-stimulated insulin secretion that collaborate to promote sustained hyperglycemia in diabetics. High-dose biotin appears to have the potential to rectify this situation to some degree. Presumably as a result of these effects, studies in rodent models of diabetes, as well as some pilot clinical trials in both types of diabetes, report that high-dose biotin can improve glycemic control [220,221,224,227,228,229].

With respect to the possible impact of biotin on diabetic complications, there are clinical case reports of improvements in diabetic neuropathy in diabetics using 10 mg biotin daily [230]. Furthermore, a study in diabetic rodents administered high-dose biotin reports diminished renal fibrosis and oxidative stress [231]. In light of previous rodent studies showing suppression of diabetic complications with other agents that activate sGC and with PDE5 inhibitors, it is reasonable to presume that intakes of biotin sufficient to achieve systemic activation of sGC would likewise be protective in this regard.

## 6. Taurine—Does It Reverse the Inactivating Oxidation of sGC?

In rodent models of diabetes, diets enriched in taurine have shown protective effects in the range of diabetic complications: neuropathy, retinopathy, nephropathy, atherosclerosis, and cardiomyopathy [232,233]. The mechanistic basis of this protection is obscure, as taurine does not function as a scavenging antioxidant—aside from its ability to detoxify hypochlorous acid. While hypochlorous acid—a product of activated macrophages and neutrophil—could conceivably play a role in diabetic complication, little research supports this possibility at present.

However, one credible possibility is suggested by recent research. In a clinical study enrolling subjects with pre-hypertension, taurine (1.6 g daily) not only lowered blood pressure relative to placebo, but also nearly doubled serum levels of hydrogen sulfide (H_2_S) [234]. A previous study with kittens had shown that supplemental taurine increases H_2_S levels by up-regulating expression of the enzyme catalyzing its production, cystathionine gamma lyase (CGL); induction of this enzyme has also been shown in the arteries of taurine-supplemented mice [234,235]. This makes sense homeostatically, since an alternative fate of cystathionine is conversion to taurine; if taurine is not needed, a higher proportion of cystathionine can be routed to H_2_S synthesis. H_2_S has recently been reported to reactivate oxidized sGC by reducing the heme ferric iron to ferrous form [236]. Hence, if this effect is significant at physiological concentrations of H_2_S, taurine-rich diets have the potential to up-regulate NO-mediated (and presumably biotin-mediated) production of cGMP. (Furthermore, perhaps physiological activation of sGC should be viewed as a collaboration between the gases NO and H_2_S.) Additionally, H_2_S can function as a phase 2 inducer, up-regulating glutathione synthesis and the expression of various antioxidant enzymes [237]. So taurine’s impact on diabetic complications in rodents may be attributable, at least in part, to increased production of H_2_S. In diabetic rodents, H_2_S donors have exerted protective effects on development of nephropathy, neuropathy, and cardiomyopathy, while aiding wound healing [238,239,240,241,242,243]. The extent to which these benefits might reflect improved sGC function remains unclear.

Also, taurine has been shown, in vitro, to act as an agonist for the liver X receptor-alpha (LXRalpha)—albeit it does not promote lipogenesis in hepatocytes [244]. Whether this phenomenon is relevant in vivo when taurine is administered orally in feasible doses has yet to be assessed. In rodent models of diabetes, pharmaceutical agonists for LXR have been reported to have favorable effects on nephropathy, neuropathy, retinopathy, atherosclerosis, and cardiomyopathy [245,246,247,248,249,250,251,252,253].

To date, few clinical studies have evaluated taurine supplementation in diabetics. In type 1 diabetics, two weeks of taurine supplementation (1.5 g daily) was found to reverse endothelial dysfunction and arterial stiffness in conduit vessels [254]. On the other hand, 12 months of taurine supplementation (3 g per day) failed to influence renal function in type 2 diabetics (no impact on microalbuminuria or biomarkers for fibrosis) [255]. Owing to its low cost, lack of flavor, high solubility, and complete safety, taurine could readily be included in functional foods or drinks designed for use by diabetics.

## 7. Addressing the “Metabolic Memory” Phenomenon

Diabetic retinopathy and nephropathy are distinguished by the fact that, once set in progress, they often continue to progress despite an improvement in glycemic control; as noted in the Diabetes Control and Complications Trial, a marked improvement in diabetic control can slow but not stop this progression [256]. Conversely, following several years of tight glycemic control, the onset of these complications is delayed relative to that in other diabetics with comparable levels of glycated hemoglobin [257,258]. These phenomena appear to reflect a “metabolic memory”, whereby prolonged exposure to excessive glycemia over months or years causes a sustained change in the differentiation state or metabolic behavior of the microvasculature which fails to revert to normal once the inciting stimulus of hyperglycemia is substantially alleviated. Indeed, once hyperglycemia triggers oxidative stress in the microvasculature, it persists of its own accord after near-normoglycemia is restored [259,260,261]. This phenomenon has been replicated in rat models of diabetic retinopathy. Persistent epigenetic changes in DNA and histones, as well as progressive damage to mitochondrial DNA and mitochondrial dysfunction, have been demonstrated in the retinal vasculature of rats exposed to several months of hyperglycemia followed by several months of better glycemic control [262,263,264,265,266,267,268,269,270,271]. These epigenetic shifts up-regulate expression of Keap (functional antagonist of the Nrf2-mediated antioxidant phase 2 response) and down-regulate expression of the mitochondrial superoxide dismutase (SOD). High levels of advanced glycation end products (AGEs) in skin collagen are predictive of progression in retinopathy and nephropathy, independent of glycated hemoglobin level—suggestive of the possibility that AGEs in long-lived extracellular matrix proteins may be mediators of the metabolic memory phenomenon [272].

In one intriguing recent study, rats were rendered diabetic with streptozotocin injection; after 6 months of hyperglycemia, their glycemic control was markedly improved by daily administration of insulin for another 6 months. At the 5-month point, some of the rats received an intravitreous injection of a recombinant viral vector carrying the gene for the mitochondrial manganese-dependent SOD. At the end of this year-long study, the retinal microvasculature showed marked progression of retinopathy in those rats who had not received the SOD, whereas those which had were substantially protected from retinopathy [273]. This strongly suggests that, when glycemic control can be improved, concurrent measures which succeed in controlling the oxidative stress in the retinal microvasculature can be useful for controlling retinopathy—and likely reversing the associated epigenetic shifts. To what extent do up-regulated NADPH oxidase activity and uncoupled eNOS contribute to sustained retinal oxidative stress when retinopathy progresses after restoration of glycemic control? A contribution of mitochondrial oxidative stress can be deduced from the mitochondrial damage seen in this circumstance, and from the utility of mitochondrial SOD in controlling this syndrome. In cultured human endothelial cells exposed to hyperglycemia for 2 weeks and normoglycemia for a further week, markers of oxidative stress persisted during normoglycemia, but exposure of these cells during the final week to several antioxidants—lipoic acid, oxypurinol, and the NADPH oxidase inhibitor apocynin—diminished this oxidative stress [259]. These data point to a role for persistent NADPH oxidase activation in the metabolic memory phenomenon. It would be intriguing to examine the impact of the nutraceutical regimen recommended here in rodent models of persistent diabetic retinopathy. In this regard, the phase 2-inducing nutraceutical lipoic acid, alone or in conjunction with other antioxidants (including macular carotenoids), has shown some efficacy in the rat model of diabetic retinopathy [27,274].

The evident implication of the metabolic memory phenomenon is that, except in patients whose diabetes is of very recent origin, optimizing glycemic control may not be sufficient to prevent onset and progression of diabetic complication—additional measures which address the aberrant metabolism of the microvasculature are needed as well [259].

## 8. Ancillary Nutraceuticals

As suggested by the foregoing, additional antioxidants have potential for controlling diabetic complications, and presumably could be used as complements to the core nutraceutical program suggested here. Phase II inducers, via activation of the nrf2 transcription factor, boost the expression of a range of antioxidants enzymes, and also induce the enzyme that is rate-limiting for glutathione synthesis [275,276]. Lipoic acid—particularly its physiological R enantiomer, which is transported more efficiently [277]—is outstanding in this regard, as it has favorable pharmacokinetics, and has been shown to be clinically useful in management of diabetic neuropathy [278,279,280,281]. The glutathione-boosting efficacy of phase 2 inducers can be enhanced by concurrent administration of N-acetylcysteine, a delivery form for cysteine. Recent studies suggest that the elderly may have an increased requirement for dietary cysteine, as they need higher intakes of this amino acid to maintain youthful tissue glutathione levels [282,283,284,285]. Another nutraceutical, the hormone melatonin, can work like phase 2 inducers to increase the expression of a range of antioxidant enzymes and boost glutathione synthesis—albeit its efficacy reflects activation of receptors independent of nrf2 [286]. Astaxanthin, perhaps the most effective natural lipid-soluble membrane antioxidant, may have potential for suppressing mitochondrial generation of superoxide by protecting the inner membrane respiratory chain from oxidative damage; this may account for its ability to decrease the oxidative stress stemming from ischemia-reperfusion damage [287,288,289]. Beneficial effects of astaxanthin on renal, retinal, and other complications of diabetes have been reported in diabetic rodents [290,291,292,293,294,295]. The xanthophyll carotenoids, lutein and zeaxanthin, have potential for dampening retinal oxidative damage in diabetics [296,297]. In regions where soil selenium levels are low and selenium intakes are suboptimal, supplementation with modest nutritional doses of selenium, an essential cofactor for glutathione peroxidase and other antioxidant enzymes, may be helpful [298,299]. Each of these agents has shown efficacy in various rodent models of diabetic complications [290,292,300,301,302,303,304,305,306,307,308,309,310,311,312,313,314,315,316,317,318,319,320,321,322].

On the other hand, high-dose vitamin C may not be recommendable for diabetics. Labile extracellular copper appears to promote the production of advanced glycation end-products in diabetics, and an increase in plasma levels of ascorbate could be expected to make this copper more toxic by maintaining it in its reduced cuprous form [323,324]. This mechanism may explain an epidemiological study finding an increased risk for coronary events in diabetics taking high-dose, but not low-dose, vitamin C supplements; moreover, the adverse impact of labile extracellular copper may account for the ability of chelation therapy to reduce risk for coronary events in diabetics with coronary disease [324,325,326]. Conversely, high intakes of zinc, which functions as a copper antagonist via metallothionein induction, may have potential for suppression of diabetic complications [324,327,328]. Indeed, increased zinc intake has shown protective effects in rat models of diabetic microvascular disease, and, in recent Chinese epidemiology, the serum zinc levels of diabetics were found to correlate inversely with risk for nephropathy, neuropathy and retinopathy [329,330,331,332,333]. Moreover, two small clinical trials of zinc supplementation in diabetics with neuropathy have concluded that zinc can improve motor neuron conduction velocity in these patients [334,335]. A meta-analysis of controlled zinc supplementation studies in type 2 diabetics concluded that zinc can also modestly improve glycemic control [336]. Metallothionein can scavenge peroxynitrite-derived radicals [337,338,339,340], raising the possibility that high-dose zinc could promote proper coupling of eNOS. Indeed, this effect may be a mediator of the favorable impact of supplemental zinc on diabetic cardiomyopathy in rodents [339].

As noted, AGE-mediated activation of the RAGE receptor is a source of oxidative stress in diabetics. Multi-gram supplemental intakes of glycine, which can raise plasma glycine levels several-fold, have potential for suppressing formation of AGEs by competing with protein-bound lysines for formation of Schiff bases with reactive aldehydes [341,342]. When type 2 diabetics ingested 5 g of glycine four times daily for 6 months in an uncontrolled trial, glycated hemoglobin fell from a baseline level of 9.6% to 6.9%; the authors did not report fasting or post-prandial glucose in these patients, and much of this reduction may have reflected inhibition of hemoglobin glycation rather than improved glycemic control [343]. A similar effect on glycated hemoglobin was reported in glycine-treated diabetic rats [344]. In rats with streptozocin-induced diabetes, a glycine-enriched diet exerted protective effects with respect to glomerulosclerosis, cataracts, and microaneurysims of the retinal arteries [345,346,347]. Supplemental glycine also exerts anti-inflammatory effects via glycine-gated chloride channels that potentially could be of value to diabetics [348]. Since glycine is inexpensive, highly soluble, and has a pleasant sweet flavor, its utility in diabetes control should receive further clinical evaluation. No adverse effects have been reported with daily intakes of up to 20 g daily, in divided doses.

## 9. Practical Implications

PhyCB, citrulline, taurine, and high-dose folate and biotin can be expected to work in a complementary matter to control diabetic complications by getting to the root of the oxidative stress and the associated loss of NO bioactivity that play an important role in mediating these complications. PhyCB, citruline, and high-dose folate address two key sources of oxidative stress in diabetes: NAPDH oxidase and eNOS. To the extent that these fail to eliminate oxidative stress entirely, high-dose biotin can be expected to “pinch hit” for the loss of NO bioactivity by directly activating sGC. Biotin also—possibly because of its impact on cGMP production—influences enzyme induction in hepatocytes and pancreatic beta cells, and thereby can improve glycemic control. Taurine, by boosting H_2_S synthesis, may help to maintain the active reduced form of sGC. To the extent that this “core program” of nutraceutical supplementation might be suboptimally effective, it could be complemented with additional antioxidants—e.g., phase 2 inducers (e.g., lipoic acid), N-acetylcysteine, melatonin, zinc—intended to support natural cellular antioxidant mechanisms impaired by epigenetic shifts or aging. Glycine may act indirectly as an antioxidant by suppressing formation of AGEs, a key cause of oxidative stress in diabetics.

Once concentrated preparations of PhyCB are available for clinical use, it would be quite feasible to include effective doses of PhyCB, folic acid, and biotin in a single capsule or tablet. Provisionally, folate doses in the range of 10–80 mg daily, and biotin doses in the range of 10–30 mg daily, can be recommended for this purpose. The appropriate clinical dose of PhyCB remains to be defined, but extrapolation from rodent studies suggests that 100–200 mg daily might be highly effective [53]. Arguably, high-dose folate should be accompanied by a mega-dose of vitamin B12 (e.g., 1 mg/day); such an oral dose of B12 would be sufficient to correct any pre-existing deficiency of B12—even in patients with pernicious anemia—so that high folate intakes could not exacerbate the clinical course of B12 deficiency by suppressing its early symptoms (anemia) [349]. Citrulline must be supplemented in fairly high bulk (3–6 g daily) for optimal support of eNOS, so it is best administered as a powder in drinks or functional foods. Multi-gram doses of taurine likewise can be administered in this way. Until PhyCB per se is available as a nutraceutical supplement, high intakes of spirulina—preferably 15 g daily or so [53]—can be included in drinks or functional foods designed to mask its rather disagreeable taste and odor; alternatively, spirulina extracts enriched in phycocyanin can be administered in capsule form.
